# Case report: Significant relief of linezolid-induced peripheral neuropathy in a pre-XDR-TB case after acupuncture treatment

**DOI:** 10.3389/fneur.2022.985499

**Published:** 2022-09-08

**Authors:** Yuping Mo, Zhu Zhu, Jie Tan, Zhilin Liang, Jiahui Wu, Xingcheng Chen, Ming Hu, Peize Zhang, Guofang Deng, Liang Fu

**Affiliations:** ^1^Acupuncture and Physiotherapy Department, National Clinical Research Center for Infectious Disease, Shenzhen Third People's Hospital, Southern University of Science and Technology, Shenzhen, China; ^2^Breax Laboratory, PCAB Research Center of Breath and Metabolism, Beijing, China; ^3^Pulmonary Diseases Department Two, National Clinical Research Center for Infectious Disease, Shenzhen Third People's Hospital, Southern University of Science and Technology, Shenzhen, China

**Keywords:** multidrug-resistant tuberculosis, linezolid, peripheral neuropathy, acupuncture, adverse reactions

## Abstract

The revised WHO guidelines on multidrug- or rifampicin-resistant tuberculosis (MDR/RR-TB) include linezolid in the core drug group. Common adverse events of prolonged linezolid use are bone marrow suppression and peripheral neuropathy (PN). Available measures against linezolid-induced PN (LIPN) often have insignificant effects, leading to linezolid discontinuation and a decline in the success rate of MDR/RR-TB treatment. Acupuncture treatment is a symptomatic treatment measure from traditional Chinese medicine (TCM) to relieve pain with overall very low evidence and has never been reported in LIPN. The pilot use of acupuncture in a pre-extensively drug-resistant (XDR)-TB (a more severe form of MDR/RR-TB) patient exhibited significant improvements in LIPN and thus maintained linezolid in the regimen for a longer period.

## Background

According to WHO reports and guidelines, multidrug- or rifampicin-resistant tuberculosis (MDR/RR-TB) is defined as TB that is resistant to rifampicin with or without the co-occurrence of isoniazid resistance, while pre-extensively drug-resistant (XDR)-TB is TB that fulfills the definition of MDR-TB with additional resistance to any fluoroquinolones (FQs). XDR-TB means there is resistance to at least one additional group A drug in addition to pre-XDR-TB (1, 2). In group A, the oxazolidinone linezolid appears to be effective. However, linezolid is associated with considerable adverse effects (AEs), especially when used at a high dosage for a long duration (3). With the common use of linezolid in MDR/RR-TB treatment, peripheral neuropathy (PN) is likely to occur more frequently and causes long-term functional impairment that impacts the quality of life (4), against which symptomatic treatments are far from bridging the curative gap, despite the efforts being made to shorten the duration and decrease the dose of linezolid (5).

Neuropathic pain is challenging to treat due to its complex natural history, unclear etiology, and insufficient response to standard physical therapy interventions. A multimodal approach to neuropathic pain has been advocated and includes combinations of pharmacological, physical, and cognitive interventions (6). Acupuncture is a traditional Chinese medicine (TCM) treatment modality that is becoming increasingly popular around the world, and some rehabilitation professionals may be trained to provide acupuncture treatment. However, the benefits and harms of acupuncture for linezolid-induced PN (LIPN) have never been reported.

Here, we present a case of a man diagnosed with pre-XDR-TB who developed PN after 4 months of treatment with a linezolid-based regimen and received a series of acupuncture treatments.

## Case report

A 26-year-old man, who weighed 48 kg and was 183 cm, was presented to a local respiratory department in Shenzhen, China, after 4 days of fever (up to 38.8°C) and right chest pain, which was accompanied by headache, myalgia, and weakness without cough, sputum, night sweats, weight loss, cutaneous rash, diarrhea, or arthralgia. Other vital signs were within normal limits. The patient had a history of antituberculosis treatment with first-line drugs initiated in 2018 and achieved treatment success in 2019, leaving a cyst in the right upper lung lobe. There were no recent vaccinations or histories of chronic illness, except for 20 years of chronic hepatitis B with normal liver function. On physical examination, the patient appeared thin but did not have cachexia. Breath sounds in the right lung were weakened, but rales were not heard. Routine blood test results were within normal limits. Sputum and blood cultures were negative. A computed tomography (CT) scan of chest of patient showed an enlargement of the cyst, the emergence of a liquid plane in it, and patchy lesions around it. Antibiotic treatment was ineffective, and he was transferred to thoracic surgery and underwent resection of the right upper lope. However, this was not alleviated after the surgery. Further antibiotic treatment and subsequent second surgery to repair the lung were failed to control the fever, but a substantial surgery wound was left on the right chest wall that would not self-heal.

Pleural effusions were emerged after the surgery, and GeneXpert *Mycobacterium tuberculosis*/resistance to rifampicin (MTB/RIF) showed positive TB-DNA and resistance to rifampicin. The rapid molecular test was failed because of paucibacillary samples of sputum and pleural effusion. The patient came to a specialized tuberculosis hospital and received a regimen of bedaquiline, linezolid, moxifloxacin, cycloserine, and clofazimine against RR/MDR/RR-TB. Thereafter, MTB culture from bronchoalveolar lavage (BAL) and pleural effusion were positive, and the minimum inhibitory concentration (MIC) showed resistance to FQs, isoniazid, rifampicin, pyrazinamide, and ethambutol and susceptibility to bedaquiline, linezolid, cycloserine, clofazimine, protionamide, and second-line injections, which met the definition of pre-XDR-TB. Thus, we substituted moxifloxacin for protionamide. In addition to chemotherapy, the patient needed to change the dressing daily on the surgical wound of the right chest wall and was waiting for a suitable time to undergo the right chest wall repair.

After 4 months of MDR/RR-TB treatment, the patient complained of numbness and pain in the lower limbs and progressive worsening, without anemia, leukopenia/neutropenia, thrombocytopenia, diarrhea, or visual problems. The drug that was likely responsible for PN was linezolid. Notably, other markers of linezolid toxicity, such as bone marrow suppression, were absent (7). B vitamin complexes containing vitamin B6, B1, and mecobalamin; painkillers, such as pregabalin, gabapentin, and tramadol; and a subsequent reduction in the linezolid dose to 300 mg daily for weeks were ineffective in relieving the pain. By 6 months, symptoms of the patient had progressed and worsened, and the pain had spread to his legs. In his own subjective opinion, the feeling was acute, frequent disabling, and burning pain, and he also felt numbness in his legs every day, especially in the lower extremities. Continuous insomnia during the day and night was almost unbearable. His electromyography showed that the sensory conductivity of the bilateral superficial gastrocnemius muscle nerves was decelerated and that the sensory nerve action potential (SNAP) amplitude was low so that the conductivity function of the bilateral superficial gastrocnemius muscle nerves was slightly affected. Half a month later, he was diagnosed with PN, and analgesic drugs were added to alleviate the symptoms. However, the problem could not be solved.

As a last resort and after being fully informed, the patient accepted acupuncture treatment once a day at the Acupuncture and Physiotherapy Department 18 times during hospitalization. We assessed the pain pre-, post-, and during the treatment. The intensity of pain was measured with the short form of the McGill Pain Questionnaire (SF-MPQ), which includes three items (8). This questionnaire included the pain rating index (PRI), visual analog scale (VAS), and present pain intensity (PPI). The PRI is used to evaluate pain sensation and emotion, with scores of 0 (no pain), 1 (mild pain), 2 (moderate pain), and 3 (severe pain). The PPI and VAS in SF-MPQ were also used to assess overall pain status.

When evaluating item 1, we asked the patient each question and marked the corresponding level of pain according to the patient's response. For item 2, the length of the line segment in the figure was 10 cm, and the scale was determined in mm. The patient was asked to mark corresponding points on the line segment with a pen according to his own pain feeling. For item 3, we marked the corresponding score according to the subjective feelings of the patient. Finally, the PRI, VAS, and PPI were evaluated. The higher the score was, the more severe the pain was.

**Figure 1:**
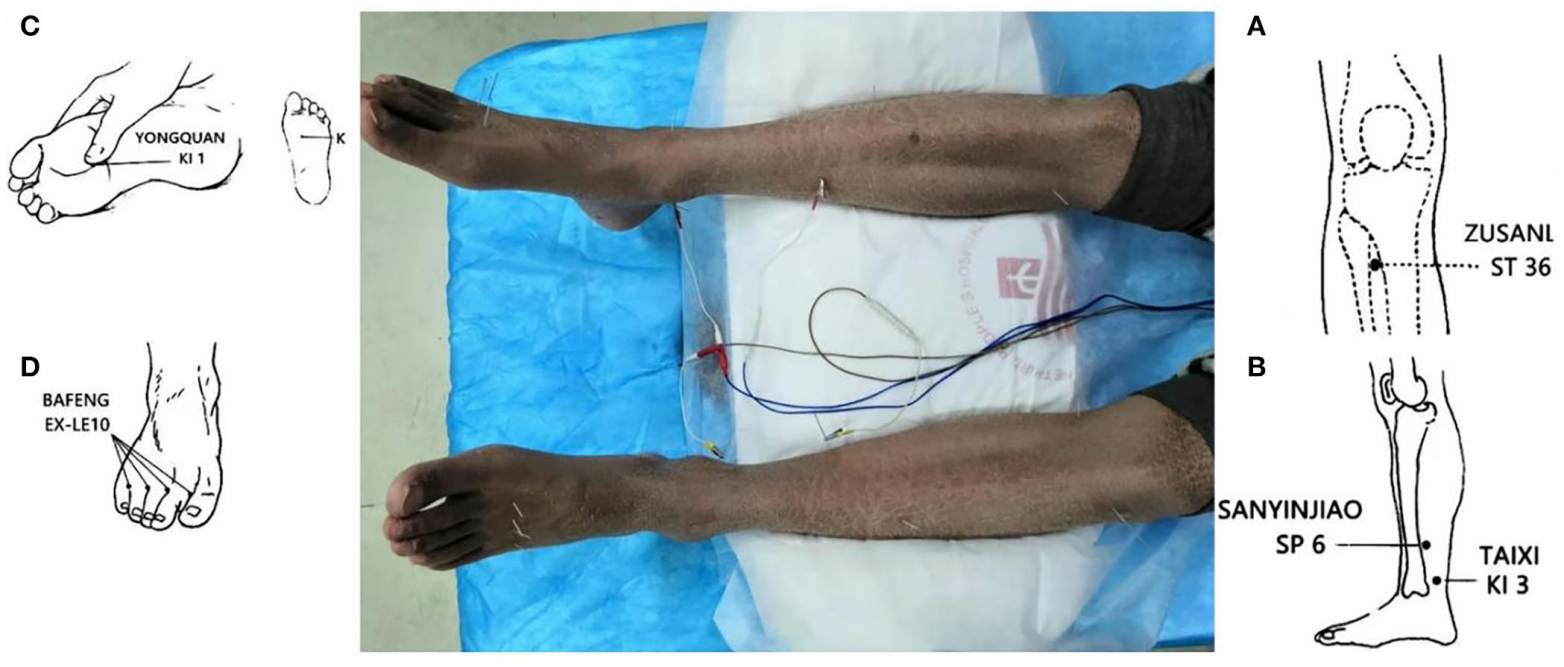
The patient on acupuncture. (1) The main acupoints for the patient, bilateral Zusanli [ST 36, **(A)**], Sanyinjiao [SP 6, **(B)**], Taixi [KI 3, **(B)**], Yongquan [KI 1, **(C)**], Bafeng [EX-LE10, **(D)**]. SP 6 and KI 3 were connected to electroacupuncture equipment for 30 min. (2) Brownish skin pigmentation and chapped skin due to prolonged use of clofazimine were observed.

A senior acupuncturist performed the therapeutic acupuncture sessions. The patient was first placed in a comfortable supine position. The acupuncture technique used to treat LIPN pain is in accordance with the “Zang-fu” and meridian system involved in the disease. The point locations for the acupuncture session are those used when treating painful conditions in the lower limbs. The main points were located in the meridians of the lower limb, such as the stomach, spleen, and kidney, for lower limb pain and the nourishing yin point and acupuncture points for the relief of pain. Subsequently, the acupuncturist used the Bilateral Zusanli (ST 36), Sanyinjiao (SP 6), Taixi (KI 3), Yongquan (KI 1), and Bafeng (EX-LE10) points (9) (Figure 1).

The acupuncturist used 0.30 × 25 mm and 0.18 × 13 mm sterile acupuncture needles (Huacheng, Beijing Keyuan Medical Device Manufactory Co., Ltd., Beijing, China). The depth of acupuncture at ST 36, SP 6, and KI 3 was 0.5–0.8 cun and that of the other points was 0.3–0.5 cun. After the insertion, the needles were manipulated to achieve Deqi (10), and the needles at SP 6 and KI 3 were connected to electroacupuncture equipment for 30 min, as shown in Figure 1. The waveform was selected as a continuous wave. Stimulation intensity was determined by the patient's endurance. The acupuncturist added TCM fumigating treatment to promote blood circulation during the acupuncture sessions.

**Figure 2:**
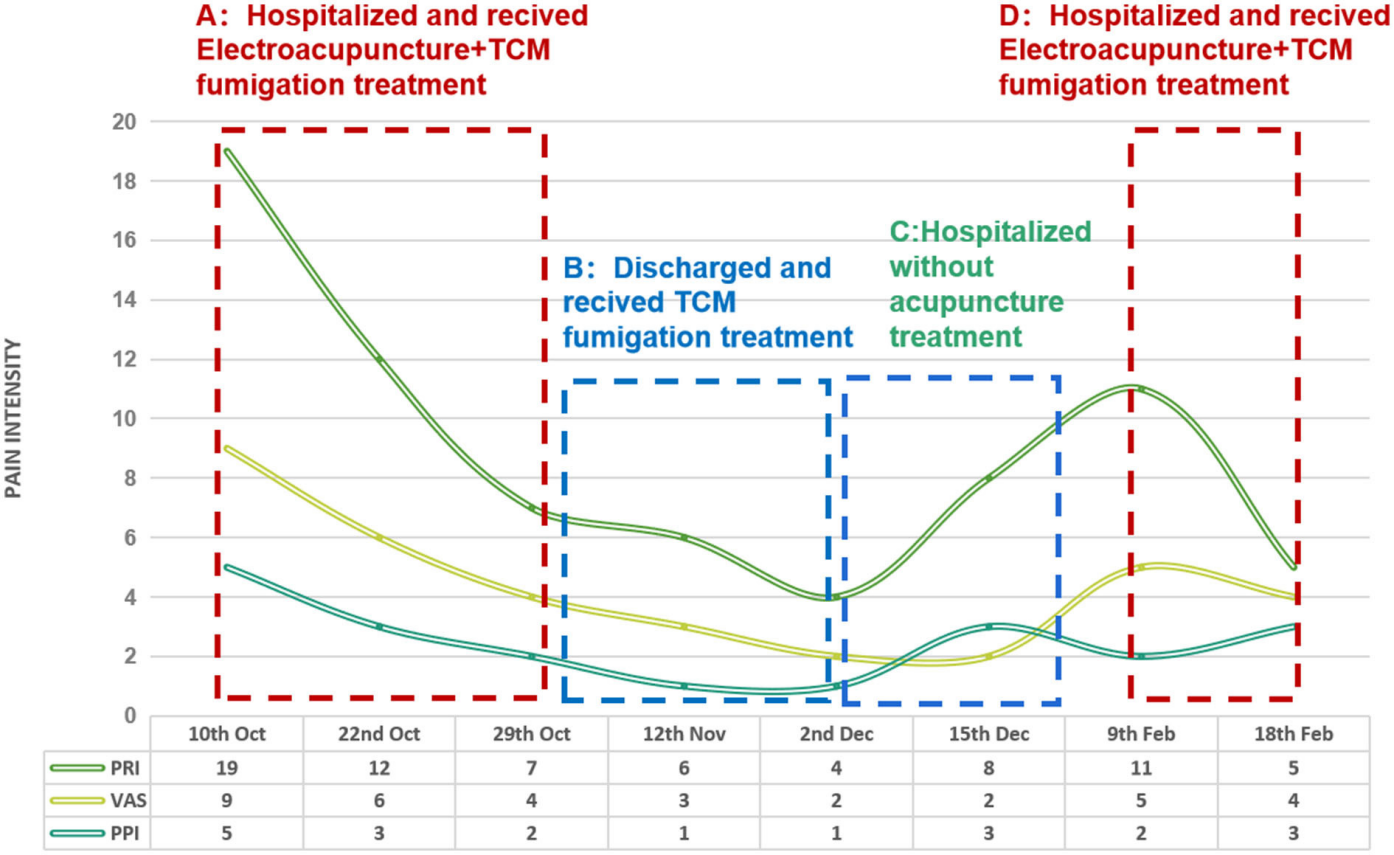
SF-MPQ Trend Chart. The evaluation process: **(A)** From 10-Oct-2021 to 29-Oct-2021, hospitalized and treated with electro-acupuncture+TCM fumigation. **(B)** 12-Nov 2021, return visit 2 weeks after discharge, TCM fumigated at home. **(C)** From 2 Dec 2021 to 15 Dec 2021, hospitalized but without acupuncture treatment. **(D)** From 9 Feb-2022 to 18 Feb-2022, hospitalized and treated again with electro-acupuncture+TCM fumigation.

After 18 rounds of treatment, the patient felt much better, indicating an important pain reduction and improved physical function, particularly in the lower limbs. There were significant changes in the scores of the SF-MPQ (PRI decreased from 19 to 7, VAS decreased from 9 to 4, and PPI decreased from 5 to 2; Figure 2). After nearly 3 weeks of treatment, he reported significant improvement in every aspect of his pain, numbness, insomnia, distress, and the reduction in painkiller intake. Thus, linezolid was increased to a dose of 600 mg daily and endured by the patient. After discharge, the patient no longer received acupuncture treatment but continued to adhere to the TCM fumigation treatment.

The PRI, VAS, and PPI scores remained low before a slight increase at 8 months after pre-XDR-TB treatment, while pleural effusion in the left chest had occurred and was drained. After a 3-month remission period until the 10th month, the patient was hospitalized again with an LIPN problem. The PRI score was increased from 8 to 11, the VAS score was increased from 2 to 5, and the PPI score was decreased from 3 to 2. After 10 acupuncture treatments and TCM fumigation, the PRI was decreased from 11 to 5, the VAS score was decreased from 5 to 4, and the PPI score did not change. The patient's lower limb pain and other symptoms were not significantly relieved, and he could not tolerate linezolid anymore; therefore, it was discontinued. Trends in pain assessment over the course of treatment are shown in Figure 2.

## Discussion

To the best of our knowledge, this is the first report on the efficacy and safety of acupuncture treatment for LIPN. This case report demonstrates that the development of PN is probably caused by linezolid treatment and its resolution following early detection and appropriate management with acupuncture. With the increasing use of linezolid for MDR/RR-TB, the management of PN in prevalent MDR/RR-TB settings is likely to be a frequent occurrence. The improvement in neuropathy following the application of acupuncture and the avoidance of immediate cessation of linezolid in our patient is encouraging.

On a larger scale, chemotherapy-induced PN (CIPN) is a common and dose-limiting toxicity that negatively affects both quality of life and disease outcomes. To date, there is no proven preventative strategy for CIPN (11). Although multiple randomized trials have evaluated a variety of pharmacologic interventions for the treatment of CIPN, no agent has shown sufficient solid beneficial evidence to be recommended for the treatment or prophylaxis of CIPN. The standard of care for CIPN includes dose reduction and/or discontinuation of chemotherapy treatment. The management of CIPN remains an important challenge, and future studies are warranted before recommendations for the use of supplements can be made (12). In the field of MDR/RR-TB treatment, when pain following LIPN is severe, a change to less effective chemotherapy agents may be needed or patients may choose to discontinue treatment. Medications used to alleviate LIPN often lack efficacy and/or have unacceptable side effects. Hence, the unmet medical need for novel treatments for the relief of this painful condition has driven our establishment of acupuncture therapy for LIPN.

In an individual patient data meta-analysis that assessed 12,030 patients treated for MDR/RR-TB, linezolid and some other agents were found to be associated with greater treatment success and reduced death (13). However, adverse events and discontinuation of linezolid were common, affecting up to 22.6% (141/624; 11 studies) of participants in the nonrandomized studies as described in a Cochrane review (3). PN usually begins in the lower limbs and is the sensory-motor axonal type. Common symptoms include pain, numbness, tingling, burning, and allodynia, usually occurring in a glove- and stocking-like distribution. While optic neuritis due to linezolid has been shown to be reversible in many instances, the reversibility of PN has been described as limited.

The patient had received linezolid for 4 months prior to developing features of PN, which gradually developed into intolerable suffering at 6 months. Three steps were taken to address the PN, but little effect was obtained. First, the prior and prolonged use of vitamin B6 for the purpose of preventing cycloserine side effects did not stop LIPN from happening, while the subsequent use of vitamin B1 and mecobalamin did not improve LIPN. A mini review on B vitamin complexes for CIPN showed that they may play a role in CIPN prevention (14), but little is known about LIPN. Based on the clinical practice in our hospital, a regional TB reference hospital, B vitamin complex has little effect on LIPN, especially those with grades 3–4 AEs. Some mild cases are gradually relieved with the help of these nutrients, but the placebo effect cannot be ruled out, while patients with moderate and severe LIPNs often cannot recover from it. Second, regarding analgesic drugs for CIPN, the best available data support a moderate recommendation for treatment with duloxetine, while the CIPN trials are inconclusive regarding tricyclic antidepressants (such as nortriptyline), gabapentin, and some other agents. Again, in our practice, gabapentin and other painkillers have shown limited efficacy for LIPN-related pain, although we did not approach LIPN exactly according to the cancer pain criteria. Third, the reduction in linezolid dosage to 300 mg daily for weeks did not reverse the LIPN pain either, indicating that medication dosage reduction has no apparent effect on immediate pain relief and therefore is not a favorable strategy for this purpose, although it may work for grades 1–2 LIPN pain in our practice. Halving the dosage of linezolid is one of the commonly used measures, but it may lead to reduced antituberculosis efficacy and an increased risk of treatment failure, not to mention the discontinuation of linezolid, especially during the intensive phase of treatment. In contrast, shortening the duration may be a favorable option. ZeNix's study explored the optimal course of linezolid, suggesting that a 2-month linezolid dosage of 600 mg daily was as effective as a 6-month dosage in pretomanid- and bedaquiline-containing regimens (15). Usually, after all the methods mentioned above were tried and failed, the only option left was to discontinue linezolid until acupuncture was introduced, at least in this case. PN has also been mentioned as a rare but possible AE of other second-line drugs, such as cycloserine and prothionamide (16), which were ultimately excluded as the cause in this case based on the response to AE management.

Acupuncture is a nonpharmacological treatment option for multiple diseases and symptoms. Although numerous studies have been performed on the efficacy of acupuncture, there have been only a few landmark high-quality randomized controlled trials (17). Acupuncture treatment is a symptomatic treatment measure to relieve pain, for which the overall quality of evidence is very low and conflicting due to study limitations (high risk of performance, detection, and attrition bias, and high risk of bias confounded by small study size) or imprecision, indicating that acupuncture was not superior to sham acupuncture or other placebos (18, 19), although it is a pain management method recommended by the Food and Drug Administration (FDA) for chronic low back pain. To date, there are no reports about acupuncture treatment for LIPN except the current one. The patient responded well to acupuncture treatment with improved SF-MPQ, sleep, and mobility scores. After treatment with acupuncture and fumigation, dose reduction and discontinuation of linezolid were avoided for a period of time. These results support the effectiveness of acupuncture as part of a multidisciplinary approach to improve LIPN pain and other symptoms.

In terms of measurements, LIPN may include numbness, pain in the extremities, and vision loss. There is no specialized scale for LIPN pain, so we used a commonly used scale for adult pain, the SF-MPQ, and other methods. For numbness descriptions, we lack an assessment scale, and the Michigan Neuropathy Screening Instrument (MNSI) can be referenced in some circumstances (20), although numbness was insignificant in this case. Vision loss can be assessed by an ophthalmologist or self-measurement, with an absence of vision loss in this case.

With regard to the choice of acupuncture sites, in TCM theory, pulmonary tuberculosis is a chronic wasting disease with the weakness of healthy Qi, the infection of MTB, and the erosion of lungs, influencing each other (21, 22). TB is the cause, and vital Qi deficiency is the basis (23). TB is located mainly in the lung and is most closely related to the spleen and kidney. According to the five-element theory for acupuncture in TCM, the lung will affect other organs, such as the spleen and the kidney, when it is in poor condition (24, 25). In this case, because the patient had no symptoms, such as cough or phlegm, when he came to our department, the acupoints of the lung meridian were temporarily not chosen, and we selected SP 6 in Spleen Meridian of Foot-Taiyin, KI 1 and KI 3 in Kidney Meridian of Foot-Shaoyin to nourish yin, invigorate kidney, and strengthen the spleen, followed by ST 36 in Stomach Meridian of Foot-Yangming to enhance health, and finally SP 6 and KI 3 were connected to electroacupuncture to increase the stimulation intensity to achieve the optimal effect. Additionally, EX-LE10 was selected to dredge meridians and collaterals and relieve pain. These acupoints were chosen on the basis of experience. Numbness and pain, as the clinical manifestations of PN, were considered to belong to the category of “arthromyodynia” in TCM, which was associated with the body's “deficiency and blood stasis,” namely, blood deficiency-induced numbness and malnutrition-induced pain. Therefore, acupuncture could be combined with TCM fumigation to promote blood circulation, remove blood stasis, and warm meridians and collaterals (26).

There are several limitations of this case report. First, the mechanistic basis of CIPN is not entirely clear (27), so the mechanism of acupuncture for relieving the relevant symptoms is also unclear. Instead of targeting the underlying pathological mechanism responsible for LIPN, we addressed the LIPN symptoms themselves (11). There are several defined mechanisms of nerve damage that take place along different areas of the peripheral nervous system, and in the near future, we plan to examine the basic science and pathobiology of LIPN (28, 29), such as whether acupuncture can promote the recovery of nerve injury. Second, for measurement, symptoms and feelings were described or scored with scales from relevant fields. In the next step, it would be better to develop a feasible scoring scale for LIPN, which requires accumulation of a large number of cases and clinical verification. In addition, through follow-up, we found that the patient's symptoms would recur if linezolid was maintained and acupuncture was discontinued. Given the grades 3–4 aggravating AEs in this case, only short-term efficacy was observed before linezolid was ultimately withdrawn, while long-term efficacy, such as MDR/RR-TB treatment success and recurrence, of the intervention of acupuncture still needs to be observed. It is also interesting to observe whether acupuncture performs better for those left with LIPN after treatment completion.

Acupuncture treatment prolonged the availability of linezolid and gained time for antituberculosis treatment, which is of great value for this patient with pre-XDR-TB awaiting chest wall reconstruction. Before recommendations for MDR/RR-TB regimens suggest shorter durations or decreased dosages of linezolid or alternative agents to replace linezolid (30), acupuncture to treat LIPN may have certain applicability in some scenarios.

## Conclusion

The common presence of PN with prolonged linezolid-containing regimens against MDR/RR-TB and the ineffectiveness of current adjunctive symptomatic treatments should alert physicians to strive for optimal AE control to minimize the risk of linezolid discontinuation and for optimization of the chances of recovery from MDR/RR-TB. In this pre-XDR-TB case, the pilot application of acupuncture from TCM ensured appropriate management to reduce chronic painful neuropathy and avoid immediate cessation of linezolid, encouraging further explorations of the indications and optimization of acupuncture treatment, given the lack of effective LIPN interventions worldwide.

## Data availability statement

The original contributions presented in the study are included in the article/supplementary material, further inquiries can be directed to the corresponding author.

## Ethics statement

Ethical review and approval was not required for the study on human participants in accordance with the local legislation and institutional requirements. The patients/participants provided their written informed consent to participate in this study. Written informed consent was obtained from the individual(s) and/or minor(s)' legal guardian/next of kin for the publication of any potentially identifiable images or data included in this article.

## Author contributions

The manuscript was drafted and substantially revised by YPM and LF. Inform consent and registration was prepared by ZZ. Acupuncture treatments were administered by YPM, JT, JHW, XCC, and MH. ZZ is responsible for outcome assessment. LF, PZZ, and GFD sought funding, diagnosed and treated primary disease, and observed the effects of the treatments. All authors contributed to the article and approved the submitted version.

## Funding

This work was supported by the National Natural Science Foundation of China (No. 82070016), the National Key Research and Development Plan (Nos. 2020YFA0907200 and 2019YFC0840602), the Guangdong Foundation for Basic and Applied Basic Research (No. 2019B1515120041), the Guangdong Science and Technology Plan (No. 2020B1111170014), the Shenzhen Scientific and Technological Foundation (Nos. KCXFZ202002011007083 and JCYJ20180228162112889), Summit Plan for Foshan High-level Hospital Construction (No. FSSYKF-2020001), and Clinical Research Plan of SHDC (No. SHDC2020CR1011B).

## Conflict of interest

The authors declare that the research was conducted in the absence of any commercial or financial relationships that could be construed as a potential conflict of interest.

## Publisher's note

All claims expressed in this article are solely those of the authors and do not necessarily represent those of their affiliated organizations, or those of the publisher, the editors and the reviewers. Any product that may be evaluated in this article, or claim that may be made by its manufacturer, is not guaranteed or endorsed by the publisher.
